# Partus Siccus

**Published:** 1844-10

**Authors:** 


*Partus Siccus.*—A case of this is detailed by M. Matthysen, in which the patient was delivered of her first child after a quick labor, but in which no discharge of liquor amnii either preceded or followed its birth. The placenta came away naturally, the uterus contracted well, and though the lochiæ were exceedingly scanty, no accident occurred in the puerperal state. The child was born alive, full grown, but extremely thin, and its skin was covered by a thick coating of vernix caseosa. [M. Matthysen erroneously supposes this to be the only case of the kind on record; instead of which many cases have been related since attention was first called to it by Rudolphi.]—*Ibid.*

